# Systematic review of birth cohort studies in South East Asia and Eastern Mediterranean regions

**Published:** 2011-06

**Authors:** Rachel McKinnon, Harry Campbell

**Affiliations:** Centre for Population Health Sciences and Global Health Academy, University of Edinburgh, Scotland, UK

## Abstract

**Background:**

Few longitudinal studies of children have taken place in the developing world, despite child mortality being concentrated there. This review summarises the methodologies and main outcomes of longitudinal studies of pre-school children (0 to 59 months) in the World Health Organization’s South East Asia (SEA) and Eastern Mediterranean (EM) Regions.

**Methods:**

A systematic search of literature using pre-defined criteria revealed 7863 papers. After application of quality criteria, 120 studies were selected for analysis.

**Results:**

The search revealed 83 studies in the SEA region and 37 in the EM region, of which 92 were community-based and 8 facility-based. Objectives were diverse but topics included growth (n = 49 studies), mortality (n = 28), nutrition (n = 24), and infectious diseases (n = 33). Only 12 studies focused on non-communicable diseases. Duration ranged from 7 to 384 months. Measurements included anthropometric (n = 56 studies), socioeconomic (n = 50) and biological sampling (n = 25), but only one study was DNA-based.

**Conclusion:**

Biobanks have emerged as the most successful approach to generating knowledge about disease causes and mechanisms. Little of this is possible to undertake in the in SEA or EM regions, however. Further longitudinal studies of young children with DNA sampling should be set up to better understand determinants of diseases in low-income countries.

Very few longitudinal studies of children have been conducted in the developing world, despite the global burden of child morbidity and mortality being centred there (1,2). A history of birth cohort studies in the UK has outlined the important determinants of individual well-being and established these studies as invaluable in planning services (3-7). Examining genetic and environmental determinants of child health and disease is fundamental in reducing child mortality, to achieve the Millennium Development Goal 4 – “to reduce child mortality by two thirds by 2015” (8), Health determinants in low and middle-income countries are somewhat different to those studied in the UK and developed world, however. The most taxing diseases in the developing world are those that affect mothers and children and continue to cause high morbidity and mortality (9). Infections are still of greatest concern (including acute respiratory infections, diarrhoea, malaria, HIV/AIDS, tuberculosis and maternal tetanus), followed by neonatal issues (including birth asphyxia or preterm birth) (2,9,10). Further birth cohort studies may better establish the determinants of such diseases and inform health expenditures. Understanding the epidemiology of those diseases has important policy implications. There has been some concern within the scientific community for example, toward the movements against a HiB-vaccine programme in India (11).

A birth cohort study with genetic samples could considerably advance the understanding of the influence of genetics and epigenetics on disease burden, particularly in developing countries. This is because the natural selection imposed by infectious diseases through child mortality has been shaping the human genome for hundreds of thousands of years (12). Large biobanks have emerged as the most successful way of harnessing the new health research technologies available and generating new knowledge about disease causes and mechanisms. Moreover, storage of samples may enable testing of future hypotheses, although the screening of biobanks for research purposes does not need to be hypothesis-driven, and the whole of the genome may be screened in search for genetic associations. There has been a surge in global interest towards birth cohort studies in recent years, as shown by the trend in global publications (**Figure 1**). It is essential however that this interest be directed toward those in greatest need in the future, thus reducing inequalities in global health research.

**Figure 1:**
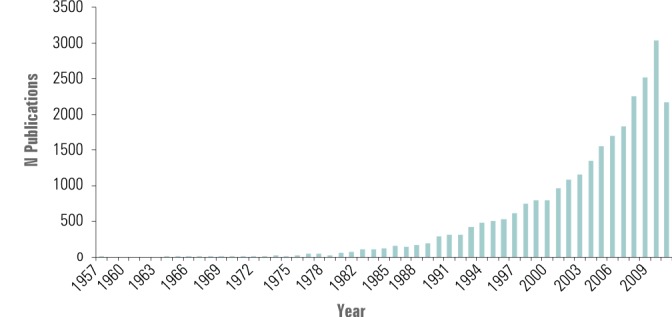
Global number of publications per year when ‘birth* cohort*’ was used as a search term in ISI Web of Knowledge database on 27 September 2010; no language or other restrictions.

The aims of the present paper were:

1. to produce a systematic review of birth cohort studies from the World Health Organisation’s South East Asia (SEA) and Eastern Mediterranean (EM) regions;

2. to examine the methodology, data collected from, and strengths / weaknesses of these studies and

3. to offer recommendations on the feasibility and sustainability of this area of research based on the findings of this review and in the context of the current literature.

## METHODS

### Search strategy

**Figure 2:**
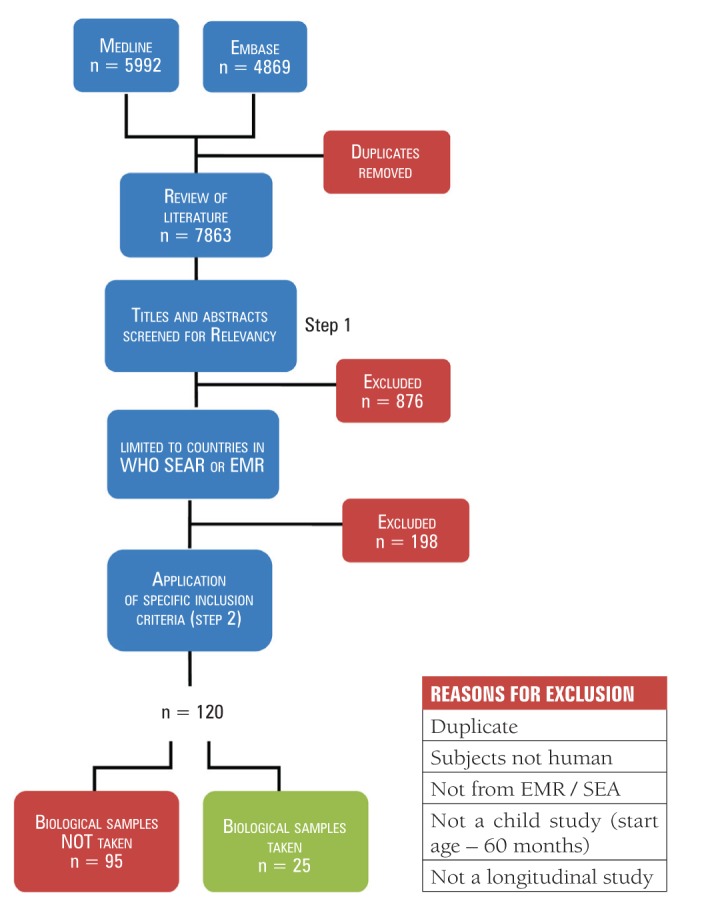
Summary of the literature search. SEAR – South East Asian Region, EMR – Eastern Mediterranean Region of the World Health Organisation (WHO).

An initial scoping exercise was conducted to identify key words and MeSH headings, and the final search terms agreed with a librarian (**Table 1**). Systematic searches were run across the following electronic databases via Ovid (**Figure 2**): Medline (1950-onwards) on 30 July 2010 and Embase (1980-onwards) on 7 July 2010.

**Table 1:** Search strategy for Medline and Embase*

**Birth Cohort / Longitudinal Study** 1. Longitudinal Studies/ 2. (birth* adj3 longitudinal).ti,ab. 3. (birth* adj3 cohort*).ti,ab. 4. (pregnan* adj3 cohort*).ti,ab. 5. famil* cohort*.ti,ab. 6. cohort* survey*.ti,ab. 7. panel stud*.ti,ab. 8. panel survey*.ti,ab.
AND
**Developing Country** 1. Developing Countries/ 2. low income countr*.tw. 3. middle income countr*.tw. 4. (low adj2 middle income countr*).tw. 5. africa/ or africa, northern/ or algeria/ or egypt/ or libya/ or morocco/ or tunisia/ or “africa south of the sahara”/ or africa, central/ or cameroon/ or central african republic/ or chad/ or congo/ or “democratic republic of the congo”/ or equatorial guinea/ or gabon/ or africa, eastern/ or burundi/ or djibouti/ or eritrea/ or ethiopia/ or kenya/ or rwanda/ or somalia/ or sudan/ or tanzania/ or uganda/ or africa, southern/ or angola/ or botswana/ or lesotho/ or malawi/ or mozambique/ or namibia/ or south africa/ or swaziland/ or zambia/ or zimbabwe/ or africa, western/ or benin/ or burkina faso/ or cape verde/ or cote d'ivoire/ or gambia/ or ghana/ or guinea/ or guinea-bissau/ or liberia/ or mali/ or mauritania/ or niger/ or nigeria/ or senegal/ or sierra leone/ or togo/ or “antigua and barbuda”/ or cuba/ or dominica/ or dominican republic/ or grenada/ or guadeloupe/ or haiti/ or jamaica/ or “saint kitts and nevis”/ or saint lucia/ or “saint vincent and the grenadines”/ or central america/ or belize/ or costa rica/ or el salvador/ or guatemala/ or honduras/ or nicaragua/ or panama/ or panama canal zone/ or mexico/ or argentina/ or bolivia/ or brazil/ or chile/ or colombia/ or ecuador/ or guyana/ or paraguay/ or peru/ or suriname/ or uruguay/ or venezuela/ or asia, central/ or kazakhstan/ or kyrgyzstan/ or tajikistan/ or turkmenistan/ or uzbekistan/ or cambodia/ or east timor/ or indonesia/ or laos/ or malaysia/ or myanmar/ or philippines/ or thailand/ or vietnam/ or asia, western/ or bangladesh/ or bhutan/ or india/ or sikkim/ or afghanistan/ or iran/ or iraq/ or jordan/ or lebanon/ or syria/ or turkey/ or yemen/ or nepal/ or pakistan/ or sri lanka/ or exp china/ or exp japan/ or korea/ or “democratic people's republic of korea”/ or “republic of korea”/ or mongolia/ or albania/ or lithuania/ or bosnia-herzegovina/ or bulgaria/ or byelarus/ or “macedonia (republic)”/ or moldova/ or montenegro/ or romania/ or russia/ or serbia/ or ukraine/ or yugoslavia/ or exp transcaucasia/ or armenia/ or azerbaijan/ or “georgia (republic)”/ or comoros/ or madagascar/ or mauritius/ or seychelles/ or fiji/ or papua new guinea/ or vanuatu/ or palau/ or samoa/ or tonga/

*Within a box, all terms were combined with Boolean operator OR.

Specific countries were those identified by the World Bank list of economies (July 2011) as being lower or middle income countries. There were no additional limitations placed on publication date, type or language.

### Inclusion and exclusion criteria

All titles, abstracts, then full papers were screened for relevance (**Table 2**).

**Table 2:** Inclusion steps

Inclusion criteria	Exclusion criteria
**Stage 1 – Titles** • Primary data OR methods from a birth cohort study / child longitudinal study (including cross-sectional data) • Meta-analysis of several birth cohort / child longitudinal studies	**Stage 1** • Not from a developing country (12)
**Stage 2 – Abstracts and Full Papers** • Country in WHO South East Asian or Eastern Mediterranean regions.	**Stage 2** • Start age <60 mo.

#### Data extraction

Data were extracted from abstracts and entered into Microsoft Excel sheets. Where more than one paper referred to a single study, the first chronologically published paper was entered into the database and any additional data from other paper(s) imputed into relevant cells. Noted study characteristics include: country, city/state, urban/rural, year of start, recruitment strategy, start number, start age, proportion of population, specific population characteristics, measurements and observations, measurement frequency, family inclusion, end age, duration, attrition, study aims, funding.

#### Data analysis

Measurements were categorized into anthropometric, socioeconomic, and biological samples / measurements. Study aims and/or outcomes were characterised into topics. These included infectious diseases (respiratory tract infections (RTIs), diarrhoea, other), non-communicable diseases, nutrition, growth, socioeconomic factors, and mortality. Some studies were relevant to ≥1 topic. To provide an overview of studies, data types were counted. Where possible, summations and calculations were done with Excel formulae to minimise human error. A specialist programme (StatPlanet MapMaker, developed by Frank van Cappelle, 2011; http://www.sacmeq.org/statplanet) was used to display the geographical spread of studies.

## RESULTS

### Study characteristics

**Figure 3:**
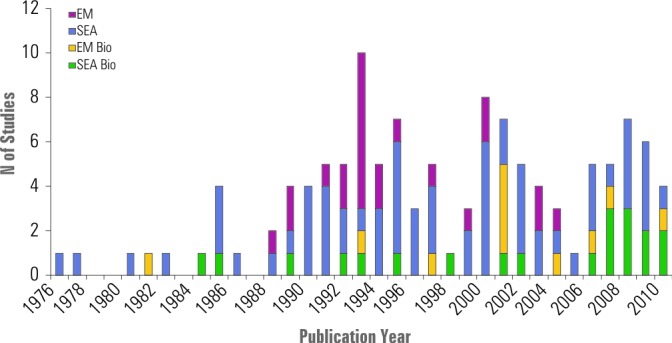
Number of studies published each year (n = 120). EM – Eastern Mediterranean, SEA – South East Asia, Bio – countries in the region taking biological samples (Bio).

**Figure 4:**
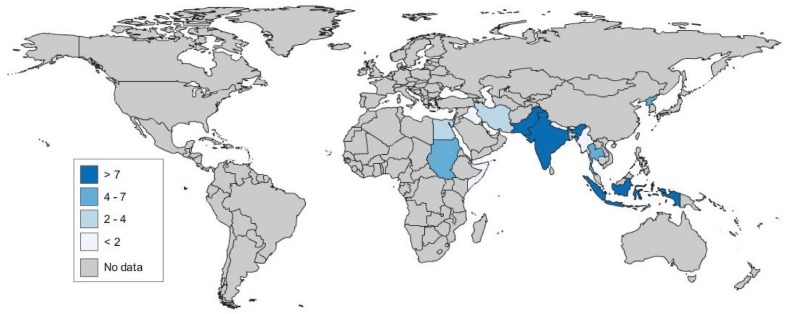
Geographical spread of all birth cohort studies in South East Asia (SEA) and Eastern Mediterranean (EM) regions (n = 120).

We analyzed 120 studies. There were only a small number of papers being published each year on birth cohort studies in the SEA or EM regions (with a maximum of 10 in total) and little change in trend in the past 20 years (**Figure 3**). In total, there were more than twice the number of studies in the SEA region (n = 83) compared to the EM region (n = 37) and these are most concentrated in India and Bangladesh and in Pakistan, respectively (**Figure 4**).

An overview of studies (**Table 3**, **Supplementary Web Table 1**)(Supplementary Web Table 1) revealed large diversity in their methodologies.

**Table 3:** Characteristics of all studies in South East Asia (SEA) and Eastern Mediterranean (EM) regions (n = 120)

Characteristic		WHO SEA	WHO EMR	EMR or SEA (% 120)
Year	Pre 1980	1	1	2 (1.7)
	1980–1989	9	5	14 (11.7)
	1990–1999	31	16	47 (39.2)
	2000–2010	42	15	57 (47.5)
Number of subjects at start	<100	4	2	6 (4.2)
	100–499	25	6	31 (25.8)
	500–999	13	5	18 (15.0)
	≥1000	22	11	33 (27.5)
	No data*	19	13	32 (26.7)
Max age at start (months)	<0	5	24	29 (24.2)
	0–3	28	1	29 (24.2)
	3–6	0	0	0 (0.0)
	6–12	1	0	1 (0.0)
	12–59	14	1	15 (12.5)
	No data*	35	11	46 (38.3)
Duration of follow up (months)	<12	1	0	1 (0.8)
	12– 24	16	13	29 (24.2)
	25–60	15	3	18 (15.0)
	>60	3	0	3 (2.5)
	No data*	48	21	69 (57.5)
Measurements / Samples (≥1 possible per study)	Anthropometric	40	16	56 (46.7)
	Socioeconomic	28	22	50 (41.7)
	Biological	17	8	25 (20.8)
	DNA	0	1	1 (0.0)
Family included*	Yes	11	7	18 (15.0)
	No	40	13	53 (44.2)
	No data*	32	17	49 (40.8)
Setting	Community-based	65	27	92 (76.6)
	Facility-based	5	3	8 (6.6)
	No data	13	7	20 (16.7)
Topic (≥1 possible per study)	Growth	31	18	49 (40.8)
	Nutrition	21	3	24 (20.0)
	Infectious diseases	24	9	33 (27.5)
	Non-communicable diseases	6	6	12 (10.0)
	Socioeconomic effects	13	7	20 (16.7)
Mortality		25	3	28 (23.3)

*Some papers did not include data searched for. Moreover, some full papers (n=22) were not obtained and details not presented in abstract.

**Figure 5:**
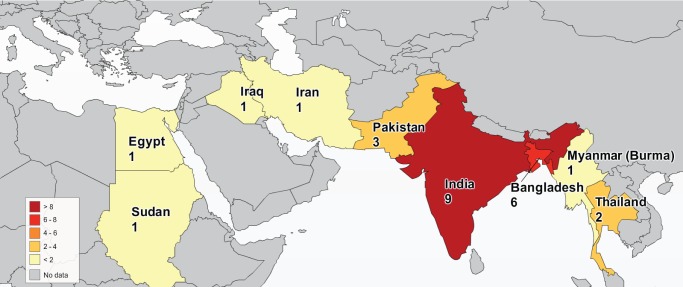
Geographical spread of birth cohort studies in the Eastern Mediterranean (EM) and South East Asia (SEA) regions taking biological samples (n = 25).

The number of children enrolled ranged from 22 (13) to 5 711 337 (14). The latter were recruited retrospectively in Korea using nationally linked birth and death certificates, but made no active measurements. The largest prospective study was a cohort of 3729 in rural Bangladesh followed from birth for 36 months to examine the effect of birth spacing on mortality (15). In most studies (58), subjects were <3 months of age at the start, with many studies recruiting mothers during pregnancy. A number of the studies presented recruited older children (**Table 3**, **Supplementary Web Table 1**)(Supplementary Web Table 1). Studies tended to be of short duration (12–24 months), but at least three of them followed up their subjects for >60 months. The Mysore Parthenon cohort (16), in which maternal serum folate, B12 and homocysteine concentrations were measured during pregnancy and child cognition (measured at 9 years) was a positive example. An even better established cohort is a New-Delhi cohort contacted at mean 32 years, with birth weight and adult glucose metabolism examined (17). Most studies tended to record socioeconomic measurements. These included parental age, education and occupation, and often indicators of household wealth (including monthly income, land size, possession of animals, and possession of transportation). Living circumstances (number of rooms: family size ratio, water supply, proximity of latrine) were also measured occasionally. Other observations commonly made in retained studies included family size and spacing and nutrition practices (prelactal feeds, exclusivity of breastfeeding, specific supplements given). Finally, exposures (eg, specific occupational hazards, household cigarettes or fuel-burning), utilisation of health services and mental health were also noted. There were only 25 studies taking biological samples (**Figure 5**) and their characteristics are presented in **Tables 4****–****5**.

**Table 4:** Studies in South East Asia (SEA) and Eastern Mediterranean (EM) regions taking biological samples (n = 25)

Ref	Author	Country	Location	Publication year	Study type	Number subjects enrolled (attrition %)	Max age at enrolment (months)	Duration of follow up (months)	Frequency of follow up
58	Bhuiyan et al.	Bangladesh	Dhaka	2009	prospective	238 (25.9)	0	24	Monthly
34	Rousham et al.	Bangladesh	Jalampur	1998	prospective	117 (4.9)	59	12	0, 2, 4, 6, 8, 12 months
61	Black et al.	Bangladesh	Matlab	1984	prospective	197 (–*)	48	12	Every second day
129	Raqib et al.	Bangladesh	Matlab	2007	retrospective	132 (0)	0	60	0, 60 months
35	Hasan et al.	Bangladesh	Mirzapur	2006	prospective	252 (3.2)	0	24	Twice weekly
77	Granat et al.	Bangladesh	Svar	2007	prospective	99 (3.0)	0	24	Fortnightly
55	Bhan et al.	India	Anapur-Palla	1989	prospective	452 (0)	35	13	Weekly
17	Fall et al.	India	Dehli	2008	retrospective	1492 (0)	0	384	6 monthly
76	Gladstone et al.	India	Haryana	2007	prospective	281 (7.3)	10	20	Weekly
119	Mathur et al.	India	Hyderabad	1985	prospective	721 (–*)	59	12	*
63	Broor et al.	India	Mysore	2010	cross-sectional	663 (19.2)	<0	120	Annually till 5 years, 6 months thereafter
146	Yajnik et al.	India	Pune	1995	retrospective	379 (–*)	0	48	*
68	Coles et al.	India	Tamilnadu	2001	prospective	539 (13.9)	0	6	2 monthly
51	Banerjee et al.	India	Vellore	2006	prospective	452 (–*)	0	36	Twice weekly
126	Raghupathy et al.	India	Vellore	2010	retrospective	2218 (13.8)	0	336	0, 3 mo, every 6 months until 14 year, mean 336 months
100	Khin-Maung-U et al.	Myanmar	Intakaw	1990	prospective	75 (0)	59	6	Monthly
148	Burke et al.	Thailand	Bangkok	1988	retrospective	218† (–*)	48	8	Daily
132	Ruangkan-Chanasetr et al.	Thailand	Bangkok	2002	prospective	84 (–*)	0	72	Twice yearly till 2 years, 6 years
149	Bassily et al.	Egypt	Alexandria	1999	prospective	169 (–*)	<0	18	At 8, 18 months
98	Kelishadi et al.	Iran	Isfahan	2007	prospective	442 (0)	0	0, samples stored for future	At birth, 72h
13	Amin-Zak et al.	Iraq	Baghdad	1981	retrospective	32 (0)	12	66	At 18, 42, 66 months
99	Khalil et al.	Pakistan	Lahore	1991	prospective	1476 (–*)	0	24	*
115	Lone et al.	Pakistan	Lahore	2004	prospective	629 (–*)	<0	1	At 1 months
142	Tikmani et al.	Pakistan	Karachi	2010	prospective	1690 (–*)	0	2	Weekly
88	Ibrahim et al.	Sudan	Khartoum	2006	prospective	205 (16.1)	0	24	6 monthly

*Not stated.

†Branch (aged 4 years) of a larger study (aged 4 – 16 years).

**Table 5:** An overview of studies taking blood samples

Reference	Blood sample			Analysis	Frequency
	Subject	Cord	Maternal		
58	Y	Y	N	ELISA – ABO & Rh group; Anti-H Pylori IgA and IgG. (Samples stored at –70°C)	Every 3 months
34	Y (fi ngerprick)	N	N	Serum albumin, IgA, alpha-1-antichymotripsinogen	Every 2 months
61	Y	N	N	T-cell telomere length, CD3 concentration, plasma interleukin 7 concentration; serum stored at –70°C and PBMCs stored for DNA.	Once (60.8±3.2 months)
17	Y	N	N	Serum glucose, lipids, insulin (fasting and after oral load)	Once (25–32 years)
63	N	N	Y	Hb, B12, folate, homocysteine	Once (30±2 weeks gestation)
146	Y	N	N	Glucose (0, 30 min after oral load)	Once (48 months)
126	Y	N	N	Fasting glucose, fastinginsulin	Once (mean 28 years)
148	Y	N	N	Anti-dengue antibodies – haemagluttinin inhibition methods	At 0 and 8 months
132	Y	N	N	Serum lead	Biannually
149	Y	N	Y	Maternal anti-H. Pylori IgG in 3rd trimester; subject anti-H. Pylori (Pylori-stat ELISA)	3rd trimester, At 7–9 and 18 months
98	N	Y	N	Triglyceride, LDL-c, HDL-c, apo-A, apoB, Lpa; frozen plasma sample stored	Once (birth) (stored for future study)
13	N	N	Y	Mercury	At 0–18 months
115	N	N	Y	Hb	Once (3rd trimester)
142	Y	N	N	bilirubin	If jaundiced (assessed weekly)
88	Y (heel prick)	Y	N	ELISA and immunofluorescence for measles IgG	At 6, 12, 24 months
Total = 15	11	1	4		

Y – yes, N – no

### Biological sampling

We found a total of 25 studies in this region which took and stored biological samples (9 of them conducted in India, 6 in Bangladesh and 3 in Pakistan) (**Figure 5**). Their characteristics are shown in **Table 4**. The samples collected in a total of fifteen studies that stored blood samples are shown in **Table 5**.

## DISCUSSION

A review of birth cohort studies in the SEA and EM regions has identified some positive examples of research. There is a distinct lack of studies addressing infectious diseases however, especially respiratory tract infections (RTI). Diarrhoea and RTI are a major cause of child morbidity and yet these topics receive little proportionate funding in research (18). Furthermore, biobanking has emerged as the most efficient way of studying the mechanisms of major diseases. However, there was only a single study from the SEA and EM regions in which DNA has been stored, in contrast to the numerous studies in the developed world benefitting from the Human Genome Project (19). A new birth cohort study and biobank in the SEA or EM regions would require significant planning, but such research must be directed to these areas of high child mortality in order to ensure future equity in health research.

In addition to infections, the incidence of non-communicable diseases such as cardiovascular disease, diabetes, cancers, cognitive defects and mental illness, are also increasing in low and middle-income countries (2,10). Thus, the developed world faces a dual burden of communicable and non-communicable disease. The diseases of adulthood have known associations with risk factors in earlier life. A life-course perspective of health determinants is now well outlined with understanding being drawn from previous longitudinal studies. Data from the UK NCDS study (3) for example outlined the association between maternal smoking and low birth weight (20). Risk factors even for the same non-communicable diseases are likely to differ somewhat between developed and developing countries. Therefore, any future birth cohort studies in the developing world would be more informative than those in the developed world.

Environmental exposures may differ in addition to different cultural manifestations. Breastfeeding, for example, is more prevalent in affluent members of developed countries in contrast to an association with poor socioeconomic status in low-income countries (21). Breastfeeding in the developed world is linked to obesity; however this may be socially patterned with high income (21). Comparative trends between countries with thus the removal of confounding factors may lead to a better understanding of causality of disease. Furthermore, the influence of the environment on child development has been better explored with new technologies made available by the Human Genome Project (22). Following the association of maternal smoking with low birth weight as outlined in the NCDS study (3), specific polymorphisms and their associations with maternal smoking, birth weight and adult cardiovascular disease have been examined (23).

### Limitations

The search for studies was systematic, explicit and designed to have high sensitivity. The databases searched are extensive and thus it is unlikely that any studies of interest were missed. In the future, however, further databases could be searched, including IndMed and the Global Health Library. More studies may also be found by screening the cited papers. Due to the small number of studies, no further quality criteria were set, such as minimum number of subjects or minimum duration. This ensured a valid overview. Application of further exclusion criteria could highlight higher quality studies for analysis. Moreover, no distinction was made in this review between prospective and retrospective studies (with individually linked data from cohort at <60 months). Detailed analysis of only prospective studies may better inform the methodological considerations of a future prospective birth cohort study. In addition, analysis of more variables could be useful. Frequency of measurements, although documented for individual studies, was not compared across studies for example. One author (24) noted that more frequent anthropometric measurements resulted in faster recognition of reduced growth in infants such that therapeutic interventions (eg, food supplements) could be directed to individuals more quickly but that it resulted in more false reports of reduced growth at younger ages. Moreover, attrition data was noted for individual studies but no comparison made between studies, including for example between geographic areas (rural vs. urban), setting (community-based vs. facility-based), start number, study duration, measurement types. Again, this may be informative when planning a new study. Finally, whilst the end date of studies was noted where possible, there was often no indication in papers as to whether or not the cohort may be followed up later.

### Study quality and comparison to international birth cohort studies

A comparison with birth cohorts in other low-income regions (including Sub-Saharan Africa) would be interesting, as well as to cohorts in middle-income (25,26) and developed countries. The cohorts examined in the SEA and EM regions are generally of a far smaller start number than those in the developed world. The National Child Development Study (3), British Cohort Study (4) and Millennium Cohort Study (5) for example initially recruited 16 634, 17 287 and 18 818 babies, respectively. Studies in the SEA and EM region are also generally of shorter duration than such studies. Finally, only a single study stored DNA from index children for later analysis, and no studies took genetic material from family members. By contrast, for example, the Avon Longitudinal Study of Parents and Children (ALSPAC) in the UK included 10 000 mothers and children for with consent for genetic sample storage and future analysis. Cohorts in middle-income countries, however, including the Pelotas study (25,26) and Birth-to-Twenty (27) recruited a large number of children for genetic analysis, providing a positive example.

### Funding

Few studies have detailed their source of funding, and this should be further examined. Other publications, however have described the main sources of funding in health research (18) including governments, NGOs and private organisations.

### Recommendations for future cohort studies

Considerable planning would be needed before a birth cohort study is undertaken in SEA or EM regions. A series of papers have been published detailing the steps towards a 2012 British Birth Cohort (28,29) and this review highlights some of the considerations of a study in the SEA or EM regions. Clear aims and objectives should be agreed in order to facilitate direction and efficient methodology. Data may be used to test future hypotheses, and so as much information should be gathered as concisely as possible. The Aberdeen cohort (30) has been criticised for having no information on smoking in households, despite making detailed social observations, since this was before the association between maternal smoking and low birth weight had been established (31). The study must be feasible for staff, as well as not too invasive to subjects. Studies should be sensitive to local cultures, with adversities to certain measurements considered (32). Moreover, consent for future analysis of measurements should be obtained from subjects and family (33). In some studies (34) fingerprints of parents were used to sign documents where literacy rates were low.

A large number of examinees should be enrolled in the cohort from an early age, so that conclusions can be drawn on the whole lifecourse perspective. Pregnant mothers could be sourced through prenatal services wherever possible, or through door-to-door interviews, aided by a registry of women of child-bearing age (eg, census) if available. Attrition could be minimised by following a population with a low migration rate, since this is a major reason for loss of follow-up. Mothers may also be offered incentive, for example meals at test facility (34) or health care for the duration of the study (35). One group described possible bias created by mothers attending facilities only to seek care (34). Moreover, number at follow up could be maximised through efficient planning, for example by utilising scheduled vaccination clinics or at entry to compulsory military service (20,26). Also, care should be taken to avoid recall errors. One study team gave calendars to mothers to record daily symptoms and treatment, thus reducing recall bias (36).

Local capacity must be developed and ensured before executing such a study. Laboratory staff should receive training and local field workers should be recruited. A number of sampling types towards the building of a biobank have been described including blood, saliva, and hair (37). Suitable media and storage should be further considered. The full list of studies retained for analysis in this paper is given at the end of our reference list (38–149), and their description in **Supplementary Web Table 1**. None of the present studies have included DNA from family members. Family members can act as proxies of exposure and can help identify parent-of-origin effects and de novo mutations and can help control for population stratification effects and should be included wherever possible (31).

## Additional Material

Supplementary Web Table 1
