# Supplementary material for: Systematic review of birth cohort studies in South East Asia and Eastern Mediterranean regions
Source: J Glob Health. 2011 Jun;1(1):59–71. (PMC3484744)
Supplement: Supplementary Web Table 1 [file jogh-01-059-s001.pdf]

Supplementary Table 1. Summary of all studies (n=120) in South East Asia (SEA) and Eastern Mediterranean (EM) regions

Author	Publication Year	Country	Measurements / Samples			No. Subjects	Start Age (months)	Duration (months)
			Biological	Anthropometric	Socioeconomic			
Agnihotri, B	2008	India				1525		
Ahmed, N	1993	Bangladesh			cleanliness score	7		
Akram, D	1991	Pakistan		W, H (compared to NCHS)	all high SES	1100		
Akram, D	2000	Pakistan						
Alam, D	2000	Bangladesh		W, H		584	6-48	12
Alam, N	1995	Bangladesh			household economic status	3729	0	36
Alam, N	1998	Bangladesh			household possession valuable items		0	
Alam, N	2001	Bangladesh			household economic status	2696	0	
Amin, S.	1990	India			maternal education, SES, health service utilization			
Amin-Zaki, L	1981	Iraq	maternal blood mercury, breast-milk mercury		breastfed/non, forcefed			60
Arifeen, S	2001	Bangladesh		BW		1677	0	12
Ashraf, R	1993	Pakistan			breastfeeding initiation duration, SES	1476	0	24
Avsm, Y	1995	India						
Ayatollahi, S	2001	Iran		W, H		317	0	
Badari, S	1991	India			cultivatable land, maternal/paternal education	1920	-3-0	12
Banerjee, I	2006	India	routine samples and samples from both child and contacts if any show diarrhoeal symptoms, VP6 antigen, phylogenetic (G and P typing)			351	range	

			analysis to test for transmission					
Bassily, S	2004	India	maternal and child serum tested for H. Pylori IgG		questionnaire at enrolment: maternal age, education, SES, number pregnancies, children, planned feeding practice	169	7-9, 18	18
Baqui, A.	1993	Bangladesh	multiple antigen skin test - baseline and every 3mo	W and H monthly	Y	705	<60	12
Bavdekar, A	1994	India		W, H, head circumference, expressed as Z		164+247	0	48
Bhalla, A. K.	1999	India		mid-upper arm circumference		154	1	12
Bhan, M	1989	India	Aeromonas, Campylobacter jejuni, E. coli			452		13
Bhandari, N	2002	India				162		
Bhardwaj, N	1995	India				212	<0	12
Bhuiyan, T	2009	Bangladesh	serum IgA, serum IgG, stool antigen, ABO blood group, other enteropathogens	malnutrition		238		24
Bhutta, Z	1997	Pakistan	blood culture: Staphylococcus aureus (18%), group B Streptococci (13%), and Klebsiella pneumoniae			6784	0	
Bishai, D	2003	Bangladesh			maternal schooling, land holdings, dwelling size, number of rooms	16270	9-60	

Black, R	1984	Bangladesh	stool enteropathogens - E.coli, Shigella	W (every 2mo), H (every y)				
Bosch, AM	2008	Bangladesh		H, W				144
Broor, S	2007	India	nasopharyngeal aspirates- direct fluorescent antibody testing for respiratory syncytial virus (RSV), influenza, parainfluenza viruses and adenoviruses, and also centrifugation enhanced culture for RSV			281	0	
Brush, G	2001	Sudan			maternal employment			
Brush, G	1993	Sudan		W, H	maternal income	96	0	12
Cheung, Y	2001	Pakistan		BH, thinness, H/thinness for 6mo			0	
Chowdhury, A. K.	1982	Bangladesh		W	number of country boats	1009		28
Coles, C	2001	India	serotypes		maternal literacy, number cigarettes/day in house, colostrum, gender	464	0	
Drewett, R	1993	Thailand				60		
Dutt, D	1997	India			SES	356	0	60
Fall, CH	2008	India	glucose, lipids, insulin	H, W, BMI, waist circumference		1492	0	
Fikree, F	2000	Pakistan		W, H	water and sanitation facilities, availability of electricity, type of house construction material and average monthly income	738	0	
Fikree, F	1999	Pakistan		W, H compare to NCHS		727	0	24

Ghosh, R	2006	Bangladesh				>140		27
Ghosh, S	1976	India			maternal literacy, maternal urban stay	802	>12	
Ghosh, S	2002	India		W				
Gladstone, B	2007	India			Morbidity (community diagnosis / hospitalization / clinic visit)	452	0	19
Granat, SM	2007	Bangladesh	nasopharyngeal samples from children and adults cultured and serotyped				0	12
Gubhaju, BB	1985	Nepal			birth spacing, sibling mortality			
Hafeman, D	2007	Bangladesh			Y	3837	0	12
Hagekull, B	1993	Pakistan			SES, housing quality, area (urban/peri-urban/village), sanitary conditions, family composition	1746	0	
Harrison, GA	1994	Sudan		W, H				
Harrison, GA.	1992	Sudan		W, H		203	0	12
Hasan, K	2006	Bangladesh	Stool samples monthly and if diarrhoea		breastfeeding/nonexclusive/exclusive, number in household, animals, monthly income, water, disposal	252	0	24
Hauspie, RC	1980	India		H, compared to Indian and European standards		563		168
Hebert, JR	1985	India		W&W-for-age	food expenditure	627		
Hirve, SS	1994	India			SES; parity, birth interval	1922	<0	
Huttly, SR	1989	Bangladesh					0-48	46
Ibrahim, MM	1994	Somalia			maternal age, occupation, literacy, household size	431		12
Ibrahim, SA	2006	Sudan	cord blood, heel prick				0	24
Jahari, AB	2000	Indonesia					12, 18	12
Jalil, F	1989	Pakistan		H, W	family education, housing	910	0	24

					standards			
Jalil, F	1993	Pakistan			SES, social structure	1476	0	
Jeon, BP	1996	Korea			school type, income			
Jirapinyo, P	2005	Thailand		W, H				60
Kabir, M	1995	Bangladesh						
Kabir, Z	2002	India					0-59	96
Kabir, Z	2003	India			caste, area, family size	15578		
Kardjati, S	1991	Indonesia		BW, growth (compared NCHS)	breastfed/non, forcefed	561	0	
Karlberg, J	1993	Pakistan		H, W, W/L	3 areas: urban, periurban, rural		0	
Kelishadi, R	2007	Iran	cord blood lipid (triglyceride, apoB)	BW, PI		442	0	
Khalil, K	1993	Pakistan	stool cultures	W, H monthly till 36 months	SES	1476	0-18	60-78
Khin-Maung-U	1990	SEA			Breath-hydrogen tests were performed after a rice meal at monthly intervals 6 months on 75 children; anthropometric measurements with growth rates every 3 months		1*, 3*	H, W (compared NCHS)
Kim, J	2009	Korea			parental education, occupation (birth cert)	1,329,540	0	
Kim, TH	1988	Korea			maternal age, birth order, mother's education, number of rooms	5000		
Kirksey, A	1991	Egypt			household SES		>-3	
Koenig, MA	1990	Bangladesh				16270		44
Koenig, MA	1990	Bangladesh						
Kolsteren, PW	1997	Indonesia		BW, BH, PI, H, W			0	
Kolsteren, PW	1997	Indonesia		W, H			0	
Kongsomboon, K	2004	Thailand						
Krishnaswamy, K	2002	India		BW, adult BMI, BP		128		
Kulsoom, U	1997	Pakistan			breastfeeding initiation supplementation prelacteal; baby gender; maternal literacy; SES;	52		

					perception milk volume adequacy			
Kusin, JA	1991	Indonesia		W, H		391		
Kusin, JA.	1992	Indonesia		W, H		542	0	
Kusin, JA	1991	Indonesia		W, H		391	0	
Launer, LJ	1990	Indonesia		W	breastfeeding time as proportion of household visit (1-4)	33		
Lone, FW	2004	Pakistan	maternal Hb	BW, IUGR		629		
Mahmud, A	1993	Pakistan				1476	0	24
Mahmud, MA	1995	Egypt			latrine not in house, mud floor in the sleeping rooms, and household exposure >10 chickens, mothers' education		0	12
Mathur, R	1985	India	diarrhoeal aetiology	initial H and W		721	<60	12
Meguid, NA	2004	Egypt				350	0	36
Mehta, S	1977	India		W, H, head circumference		68		60
Mo-suwan, L	2000	Thailand		BMI	parental income	2252		60
Nagra, S. A.	1989	Pakistan		W	feeding: breast/bottle	916	0	12
Pandey, A	1996	India				200	<60	12
Paul, B	2008	India		W, H, chest circumference		126	0	
Raghupathy, P	2010	India	adult DM/IGT/insulin secretion	H,W,BMI at birth/childhood/adolescence		2218		
Rao, S	2000	India		H, W		845	0-60	24
Rao, S	2000	India		foot length, leg length, shoulder width, sitting height, height velocity	compare to well-off Indian and to British	1020		24
Raqib	2007	Bangladesh	blood (thymopoiesis, T cell turnover (telomeres length and CD3	H,W, head circumference at birth	SES	179	0	60

			concentration), percentage of lymphocytes, acute phase response (plasma interleukin 7)					
Ravikumara, M	1996	India		BW		12283		36
Rehman, AM	2009	India				452	0	
Rousham, E	1998	Bangladesh	blood, urine		SES, maternal age	123	24-60	12
Ruangkanchanasetr, S	2002	Thailand	blood lead	W,H	RF: living in Bangkok, maternal labour workers, family income, crowded family, household occupation printing / lead smelting	511	15<	
Saha, KK	2009	Bangladesh		Anthropometric indices (both WHO and NCHS reference)		1343	0	24
Saleemi, MA	2001	Pakistan		H compared to NCHS and upper-middle-class	gender, area SES	1236	0	60
Saleemi, MA	2004	Pakistan		W, H	initiation of breastfeeding, feeding of prelacteals, exclusive breastfeeding	1991	0	
Satyanarayana, K	1986	India		H, W	work history (child labourers vs. students)	~700		
Satyanarayana, L	1995	India						
Schwekendiek, D	2009	Korea		H				
Shahidullah, M.	1994	Bangladesh				2990	0	
Son, M	2006	Korea			parents' age, education and occupation	5,711,337		
Soudarssanane, M.	1992	India				8185	0	
Thitasomakul, S	2006	Thailand				599	9	
Tikmani, S	2010	Pakistan	infant plasma bilirubin if			1690	0	

			jaundiced					
Tripathy, R.	2000	India		Mean and SDs of W/A and L/A and mean Z scores for W/A, L/A and W/L calculated separately for boys and girls with reference to NCHS-WHO and BFDS data.		114	0	12
Tuntiseranee, P	1999	Thailand		BW	socioeconomic status, maternal education, maternal occupation, family income, work exposure characteristics (Karasek's job content questionnaires), antenatal care use, parity	1797	-2	
Veena, S	2010	India			SES, breastfeeding and intro supplementary, maternal age, parents' attained schooling and rural/urban residence	514	0	
Veena, S	2010	India	mother			536	-20-32	
Yajnik, C	1995	India	plasma glucose and insulin 30min after OGL	BW		379	48	
Zaman, S	1993	Pakistan		growth	paternal literacy, home cleanliness	1476	0	24
Zaman, S	1993	Pakistan			gender, area	1476	0	24
Zumrawi, F	1988	Sudan		W, H	parity, maternal income, maternal education, home ownership, own kitchen, water supply, ventilation, sewage	439	0	

BFDS – Breast Fed Pooled Data Set, BH – length at birth, BMI – bodt mass index, BP – blood pressure, BW – birth weight, H – height, IUGR – intra-uterine growth retardation, NCHS – national child health standards, PI – ponderal index, SD – standard deviation, W – weight
*Where multiple papers have been found per study, study characteristics are drawn from all papers but the author and publication date of the

first paper chronologically has been tabulated.
